# Hypothalamic substructural integrity is associated with age, sex and cognitive function across lifespan: A comparative analysis of two large population‐based cohort studies

**DOI:** 10.1002/alz.084672

**Published:** 2025-01-09

**Authors:** Peng Xu, Santiago Estrada, Rika Etteldorf, Dan Liu, Mohammad Shahid, Deborah Frueh, Martin Reuter, Monique M.B. Breteler, N. Ahmad Aziz

**Affiliations:** ^1^ Population Health Sciences, German Center for Neurodegenerative Diseases (DZNE), Bonn Germany; ^2^ AI in Medical Imaging, German Center for Neurodegenerative Diseases (DZNE), Bonn Germany; ^3^ A.A. Martinos Center for Biomedical Imaging, Massachusetts General Hospital, Boston, MA USA; ^4^ Department of Radiology, Harvard Medical School, Boston, MA USA; ^5^ Institute for Medical Biometry, Informatics and Epidemiology (IMBIE), Faculty of Medicine, University of Bonn, Bonn Germany; ^6^ Faculty of Medicine, University of Bonn, Bonn, North Rhine‐Westphalia Germany

## Abstract

**Background:**

The hypothalamus is the body’s principal homeostatic center and plays a crucial role in the modulation of cognition. However, detailed assessments of age and sex effects on hypothalamic substructural integrity and its cognitive correlates across lifespan are still lacking. Therefore, we aimed to investigate the hypothalamic structural integrity in relation to age, sex and cognitive performance across lifespan in the general population.

**Method:**

We used cross‐sectional data from the Rhineland Study (RS) (N = 5816, 55.2 ± 13.6 years, 58% women) and the UK Biobank Study (UKB) (N = 45076, 64.2 ± 7.7 years, 53% women), two large‐scale population‐based cohort studies. Volumes of hypothalamic structures were derived from 3T structural magnetic resonance images through application of a recently developed automatic parcellation procedure (FastSurfer‐HypVINN). The standardized cognitive domain scores were derived from extensive neuropsychological test batteries. We employed multivariable linear regressions to assess the age and sex effects on volumes of hypothalamic structures, and the relation between these volumes and cognitive function.

**Result:**

Mean volumes of the total hypothalamus were 1188.4 mm^3^ (SD 113.6) in RS and 1101.9 mm^3^ (SD 119.9) in UKB. With increasing age, the volumes of total, anterior and posterior hypothalamus and mammillary bodies decreased (betas between ‐1.22 to ‐0.09 mm^3^/year in RS and between ‐3.84 to ‐0.49 mm^3^/year in UKB), and of medial hypothalamus and tuberal region increased (betas between 0.38 to 0.74 mm^3^/year in RS and between 0.21 to 0.68 mm^3^/year in UKB). Volumes of all hypothalamic structures were larger in men compared to women. Larger posterior hypothalamus volumes were associated with better global cognition (b ± standard error (SE): 0.033 ± 0.014 [RS] and 0.028 ± 0.006 [UKB], both p<0.001) and total memory (0.033 ± 0.018 [RS] and 0.015 ± 0.008 [UKB], both p,0.001), while larger anterior hypothalamus volumes were related to better executive function (0.028 ± 0.019 [RS] and 0.026 ± 0.011 [UKB], both p<0.004).

**Conclusion:**

We found strong age and sex effects on hypothalamic structures, as well as robust associations between these structures and domain‐specific cognitive functions. Overall, these findings thus implicate specific hypothalamic substructures as both markers of age‐associated cognitive decline and potential targets for future interventions.

